# Novel Therapeutic Approaches to Familial HLH (Emapalumab in FHL)

**DOI:** 10.3389/fimmu.2020.608492

**Published:** 2020-12-02

**Authors:** Pietro Merli, Mattia Algeri, Stefania Gaspari, Franco Locatelli

**Affiliations:** ^1^ Department of Pediatric Hematology/Oncology, Cell and Gene Therapy, Bambino Gesù Children’s Hospital, Rome, Italy; ^2^ Department of Maternal, Infantile, and Urological Sciences, Sapienza, University of Rome, Rome, Italy

**Keywords:** emapalumab, hemophagocytic lymphohistiocytosis, primary hemophagocytic lymphohistiocytosis, interferon-γ, interferon-gammopathies

## Abstract

Primary Hemophagocytic lymphohistiocytosis (pHLH) is a rare, life-threatening, hyperinflammatory disorder, characterized by uncontrolled activation of the immune system. Mutations affecting several genes coding for proteins involved in the cytotoxicity machinery of both natural killer (NK) and T cells have been found to be responsible for the development of pHLH. So far, front-line treatment, established on the results of large international trials, is based on the use of glucocorticoids, etoposide ± cyclosporine, followed by allogeneic hematopoietic stem cell transplantation (HSCT), the sole curative treatment for the genetic forms of the disease. However, despite major efforts to improve the outcome of pHLH, many patients still experience unfavorable outcomes, as well as severe toxicities; moreover, treatment-refractory or relapsing disease is a major challenge for pediatricians/hematologists. In this article, we review the epidemiology, etiology and pathophysiology of pHLH, with a particular focus on different cytokines at the origin of the disease. The central role of interferon-γ (IFNγ) in the development and maintenance of hyperinflammation is analyzed. The value of emapalumab, a novel IFNγ-neutralizing monoclonal antibody is discussed. Available data support the use of emapalumab for treatment of pHLH patients with refractory, recurrent or progressive disease, or intolerance to conventional therapy, recently, leading to FDA approval of the drug for these indications. Additional data are needed to define the role of emapalumab in front-line treatment or in combination with other drugs.

## Introduction

Hemophagocytic lymphohistiocytosis (HLH) is a rare, life-threatening, hyperinflammatory disorder, characterized by uncontrolled activation of the immune system. Without timely diagnosis and appropriate treatment, the prognosis of children with primary HLH (pHLH) is dismal (1). In the last decades, two multicenter, international studies (promoted by the Histiocyte Society, namely HLH-94 and HLH-2004) conducted using well-defined treatment protocols, based on multi-agent treatment strategy and allogeneic hematopoietic stem cell transplantation (HSCT), have led to improvement of the outcome of patients affected by pHLH (2, 3). Front-line treatment of pHLH is nowadays based on the combination of dexamethasone and etoposide to achieve disease control for then rapidly proceeding to allogeneic HSCT, the sole curative treatment for pHLH. Intrathecal methotrexate and hydrocortisone are used in patients with pHLH for preventing/treating central nervous system (CNS) involvement. Although, approximately 80% of patients respond to these therapies and are able to then undergo HSCT, complete responses are only achieved in less than half of patients. The majority of deaths prior to HSCT appear to be due to uncontrolled disease activity.

The results of the last international protocol, HLH-2004, investigating the addition of cyclosporine-A to the combination of dexamethasone and etoposide in 369 treatment-naïve patients did not lead to significant improvement of patient outcome in comparison with the HLH-94 study where patients were given dexamethasone and etoposide for 8 weeks, with the aim to induce remission of the disease activity [overall survival (OS) was 61% at 5 years, as compared to a 5-year OS of 54% (p = n.s.) in the HLH-94 protocol] (3). Limited data on second-line therapies are available to guide physicians regarding choice of therapy for patients with refractory pHLH. Infliximab, anakinra, alemtuzumab, daclizumab, vincristine, and other therapies have been reported in a limited number of cases (4–7). A retrospective analysis on 22 children with pHLH given alemtuzumab as treatment of refractory HLH showed a 64% overall partial response, defined as at least a 25% improvement in two or more quantifiable symptoms or laboratory markers of HLH 2 weeks following drug administration (4). Notably, no patient experienced a complete response to alemtuzumab therapy; moreover, 32% and 23% of patients experienced cytomegalovirus and adenovirus viremia. The 2-year probability of OS was 61%. A multicenter, open-label, phase I/II, non-comparative, non-randomized study (NCT02472054, which has enrolled so far some 29 patients) is currently exploring the use of alemtuzumab (in association to Methylprednisolone and CSA) in pHLH patients who had not received any specific treatment prior to enrolment except steroids and CSA. Mahlaoui and colleagues reported on a retrospective single-center experience of 38 patients treated with anti-thymocyte globulins; they received a total of 45 courses of therapy (5). Main adverse events were infections, which occurred in 10 patients (22%), leading to death in four cases. Complete response (CR) was obtained in 73% of 45 courses, a partial response (PR) in 24% of 45 courses, and no response in one case. Relapse occurred in 10 patients, including CNS involvement in five, and led to death of nine patients. These studies are not fully comparable because of significant differences in: i) type of patients treated (i.e., treatment-*naïve*, relapsed/resistant patients); ii) time of follow-up; iii) type of endpoints considered (i.e., response rate, overall survival); iv) endpoint definition (e.g., in the study of Marsh and colleagues (4) PR was defined as at least a 25% improvement in two or more quantifiable symptoms or laboratory markers of HLH 2 weeks following alemtuzumab). However, altogether, the results obtained in these studies underline that new drugs, directly targeting disease mechanism must be identified if we wish to further improve the survival of patients with pHLH.

**Figure 1:**
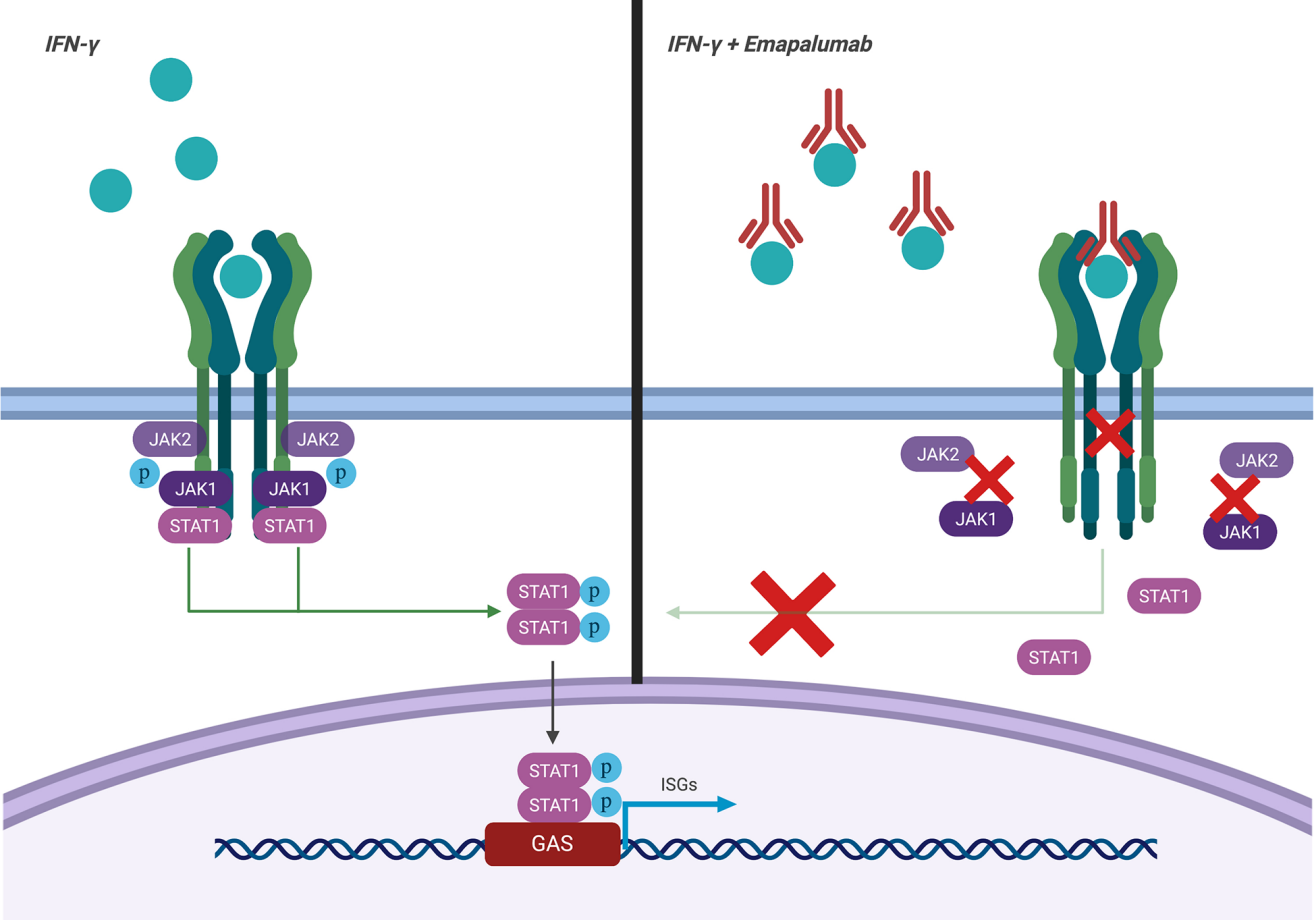
Mechanism of action of emapalumab. The drug binds to both free and receptor-bound IFNγ, inhibiting both receptor dimerization and signal transduction *via* JAK-STAT pathway.

Emapalumab-Izsg (Gamifant^®^, formerly known as NI-0501) is a fully-human IgG1 monoclonal antibody that binds with high affinity to both free interferon-γ (IFNγ) and receptor (IFNγr1)-bound IFNγ (**Figure 1**). This binding results in inhibition of receptor dimerization and signal transduction of the cytokine signalling. The drug has been approved in 2018 by the Food and Drug Administration (FDA) for adult and pediatric patients (both newborns and older children) with pHLH affected by refractory, recurrent or progressive disease or intolerant to conventional therapy (https://www.fda.gov/drugs/fda-approves-emapalumab-hemophagocytic-lymphohistiocytosis).

In this article, we will discuss the genetic background of the disease, its pathophysiology and the rationale for targeting IFNγ in patients with pHLH.

## Pathogenesis of HLH

HLH is a reactive process resulting from an uncontrolled immune response triggered by different stimuli on the background of an underlying inherited or acquired inability to eliminate the trigger itself. Three main elements cooperate in the development of the disease: a genetically-determined predisposing background, a wide spectrum of triggers and pathophysiologic cascades, which culminate in a life-threatening cytokine storm.

### The Predisposing Background and the Triggers

HLH has been traditionally divided into a primary form (i.e., pHLH), which typically manifests in children with documented genetic abnormalities of the cytotoxic function of both natural killer (NK) and T cells, and a secondary form, which tends to occur at older ages in the setting of an associated conditions, such as rheumatologic disorders, infections and malignancies, without an identifiable genetic defect. Since the first description of perforin gene mutations by Stepp et al. in 1999 (8), significant insights have been gained into the genetic mutations that predispose to the development of a pHLH phenotype. Decades of research into pHLH have revealed that the disease is predominantly due to mutations in genes crucial for the cytotoxic function of both NK cells and cytotoxic T lymphocytes (CTLs) (9, 10).

NK cells and CTLs kill infected cells by a non-secretory pathway involving Fas ligand (CD95-L), but, more importantly, through a perforin-dependent pathway. Upon activation of NK cells or CTLs, cytotoxic granules, which contain perforin and granzymes, are carried along microtubules toward the immunological synapse between effector immune cells and target cells. In this complex process, activated granules migrate, dock, and fuse with the cell membrane, releasing their contents into the synapsis (11).

In ~30% of patients with pHLH, cytolytic dysfunction is due to loss−of-function mutations in the gene encoding perforin (PRF1). When released into the immune synapsis, perforin self-polymerizes, creating pores in the plasma membrane that enable granzymes to enter the target cell and trigger apoptosis (12).

In addition to perforin deficiency, all other inherited forms of HLH in which HLH itself is the main manifestation of disease (also named familial HLH, or FHL) are characterized by failure to deliver cytotoxic granule contents (**Table 1** reports details on the different forms of the disease). Bi-allelic mutations in UNC13D (encoding Munc13-4, accounting for about one third of FHL cases), STX11 (encoding syntaxin 11, about 5% of FHL cases), and STXBP2 (encoding syntaxin-binding protein 2, also known as Munc18-2, about 10–15% of FHL cases) result into the occurrence of HLH in FHL types 3, 4, and 5, respectively (13–16). Notably, patients with STXBP2 mutations may present additional clinical features, including sensorineural hearing deficit, abnormal bleeding, and, most frequently, chronic enteropathy (17).

**Table 1:** Genetic conditions associated with predisposition to HLH.

Form	Gene (locus)	Protein	Function	Associated immunological and clinical features
***Familial HLH***				
FHL1	Unknown (9q21.3–q22?)	Unknown	Unknown	–
FHL2	PRF1 (10q21–22)	Perforin	Pore formation	–
FHL3	UNC13D (17q25)	Munc13-4	Cytolytic granule priming and fusion	–
FHL4	STX11 (6q24)	Syntaxin-11	Cytolytic granule fusion	–
FHL5	STXBP2 (19p13)	Munc18-2	Cytolytic granule fusion	Chronic enteropathy, sensorineural hearing deficit, abnormal bleeding
***Degranulation defect syndromes***				
Griscelli syndrome type 2	RAB27A (15q21)	Rab27α	Cytolytic granule docking	Partial albinism, silver-grey hair
Chediak-Higashi syndrome	LYST (1q42–43)	LYST	Cytolytic granule trafficking	Partial albinism, recurrent pyogenic infections
Hermansky-Pudlak syndrome type 2	AP3B1 (5q14.1)	AP3B1	Cytolytic granule trafficking	Partial albinism, bleeding tendency
***X-linked lymphoproliferative syndromes and EBV-susceptibility disorders***				
XLP-1	SH2D1A (Xq24–25)	SAP	Signaling in T and NK cells	Defective killing of EBV-infected B cells; May present with HLH, EBV-driven lymphoma, hypogammaglobulinemia
XLP-2	BIRC4 (Xq25)	XIAP	Inhibition of apoptosis, inflammasome control, NOD1/NOD2 signaling	Elevated IL-18, colitis, hypogammaglobulinemia
X-linked immunodeficiency with magnesium defect (XMEN)	MAGT1 (Xq21.1)	MAGT1	Mg transport, NKG2D-dependent cytotoxicity	CD4 lymphopenia, EBV-driven lymphoproliferation/lymphoma
IL-2-inducible T cell kinase deficiency	ITK (5q34)	ITK	TCR signaling	Absence of iNKT-cells, EBV-driven lymphoproliferation/lymphoma
CD27 deficiency	CD27 (12p13)	CD27	T-cell costimulatory signaling	Combined immunodeficiency, EBV-driven lymphoproliferation/lymphoma
***Autoinflammatory syndromes***				
NLRC4 gain-of-function	NLRC4 (2p22.3)	NLRC4	Inflammasome assembly	Constitutive inflammasome activation, elevated IL-1β/IL-18, recurrent fever, enteropathy
NOCARH syndrome	CDC42 (1p36.12)	CDC42	Actin assembly	Neonatal cytopenias, dyshematopoiesis, recurrent febrile episodes, urticaria-like rash

Patients with mutations in lysosomal transport (LYST or Chediak-Higashi syndrome), RAS-associated protein 27α (RAB27A or Griscelli syndrome type 2), and adaptor protein 3 B1 subunit (AP3B1 or Hermansky-Pudlak syndrome type 2) share a combination of pigment abnormalities and predisposition to develop HLH (18–20). Remarkably, each of the molecules defective in these three disorders mediates a crucial step in cytotoxic granule exocytosis at the immune synapse.

Patients with mutations in SH2D1A (X-linked lymphoproliferative disease type 1, XLP1) develop HLH after exposure to Epstein–Barr virus (EBV), and may also present with lymphoproliferative disorders or hypogammaglobulinemia (21). In XLP1, cytotoxic lymphocytes are selectively impaired in their cytotoxic response to EBV-infected B cells (22).

Some patients may have diverse genetic causes, including inflammasomopathies or even primary immune deficiencies. The importance of inflammasome activation in pHLH was recently highlighted by the identification of heterozygous gain-of-function mutations in the inflammasome component gene NLRC4 (23). Spontaneous NLRC4 activation results in over-activation of caspase-1 and excessive production of IL-18. Patients with NLRC4 mutations present with recurrent fever and severe systemic inflammation reminiscent of those encountered in HLH, which may be associated with severe enterocolitis (24). In contrast to what observed in XLP1, no defects in the cytotoxic activity of NK cells and CTLs have been identified in XLP2, which is, by contrast, caused by loss-of-function mutations in X-linked inhibitor of apoptosis (XIAP) (25). Recently, it has been hypothesized that the mechanisms underlying EBV-driven HLH in XLP2 may involve altered regulation of inflammasome and pro-inflammatory cytokine production (10). Other primary immune-deficiencies (PIDs) associated with HLH include defects in MAGT1, ITK, CD27, IKBKG, or GATA 2. In addition, HLH has been reported to occur also in patients with Wiskott–Aldrich syndrome, DiGeorge syndrome, chronic granulomatous disease, or STAT1 gain-of-function mutations (10, 26).

It is noteworthy that HLH has been observed also in patients with severe combined immunodeficiency lacking both T- and NK-cells, the two main effectors involved in the pathogenesis of pHLH. In these patients, the activation of macrophages and the subsequent cytokine storm seems to occur despite the complete absence of lymphocytes, although the precise mechanisms through which HLH develops are still obscure (26).

We and other colleagues recently described a novel hematological/auto-inflammatory condition (NOCARH syndrome) associated with the development of HLH. Affected patients shared the same *de novo* CDC42 mutation (Chr1:22417990C >T, p.R186C) and had altered hematopoietic compartment, with neonatal-onset cytopenia and dyshematopoiesis, rash, immune dysregulation, and inflammation (27).

Although secondary HLH was classically determined to have no genetic background, evidence is currently accumulating for genetic overlap between primary and secondary forms. Indeed, in some patients with classical secondary HLH, hypomorphic mutations or double heterozygosity in genes typically implicated in pHLH have been detected (28). Moreover, mutations and polymorphisms in non-cytotoxicity-related genes, affecting NK-cell receptors, cytokine production, cytokine signaling, inflammasome activation and TLR signaling, may also predispose to secondary HLH (29).

Infections and rheumatologic conditions are the most common contributors to secondary HLH. Potent triggers are viruses, especially those belonging to the herpes family. Infectious mononucleosis can mimic most features of HLH and EBV infection is the most common trigger of infection-associated HLH (26).

Besides viruses, other pathogens have been implicated as trigger for HLH, including protozoa, bacteria, and fungi (30). A frequent trigger is represented by Leishmania; diagnostic work-up of patients presenting with symptoms and signs suggestive for HLH should include the search of this pathogen (31).

HLH in autoinflammatory and autoimmune conditions is usually termed macrophage activation syndrome (MAS) (32). The highest prevalence is found in patients with systemic juvenile idiopathic arthritis (sJIA), adult-onset Still’s disease and systemic lupus erythematosus (SLE) (33). Recently, genetic studies have detected variants in MUNC13-4 among JIA patients developing MAS (34).

There is a growing recognition of malignancy-associated HLH (35). Malignancy is the most common trigger identified in adults with HLH (45%), whereas it is a relatively rare event in childhood HLH (8%). HLH may present during chemotherapy when there is typically substantial leukopenia and infectious triggers, or HLH may present at the time of diagnosis. The vast majority of triggering malignancies in adults and children are lymphomas and leukemia, most frequently T-cell derived entities, including anaplastic large cell lymphoma (ALCL) and mature T-cell lymphomas (35). Secretion of pro-inflammatory cytokines by the malignant cells could be one pathophysiological mechanism.

In addition, there is a link between pHLH and cancer: patients with XLP1 have increased risk of lymphoma and cases of malignancy have been reported in patients with FHLH 2, 4 and 5 (36, 37).

### The Cytokine Storm and the Role of IFNγ

In healthy subjects, immune homeostasis is maintained by contraction of the immune response after successful elimination of the dangerous trigger. When perforin-mediated cytotoxicity is either diminished or absent, such as in case of pHLH, severe immune dysregulation occurs as result of inefficient removal of target cells. In this respect, animal models of HLH, in which cytotoxicity-deficient mice are challenged with a virus, have been an invaluable tool for unravelling mechanism by which impairment of degranulation machinery leads to the severe perturbation of immune homeostasis typical of the disease (10, 29).

As already mentioned, a disparate group of disease triggers are possible, each of which initiates the upstream events of CD8+ T-cell hyperstimulation. In the case of pHLH, the initiation event is typically an infectious trigger. Defective elimination of infected cells drives excessive antigen presentation to CD8+ T cells, likely because of a combination of increased viral load and prolonged antigen stimulation (38). Despite that, in several large case series, an infectious trigger has been identified only in less than 50% of pHLH cases (2, 3, 39) and this percentage is even lower in cases with intrauterine or neonatal onset (40), this observation suggesting that also non-infectious stimuli may initiate the development of pHLH.

**Figure 2:**
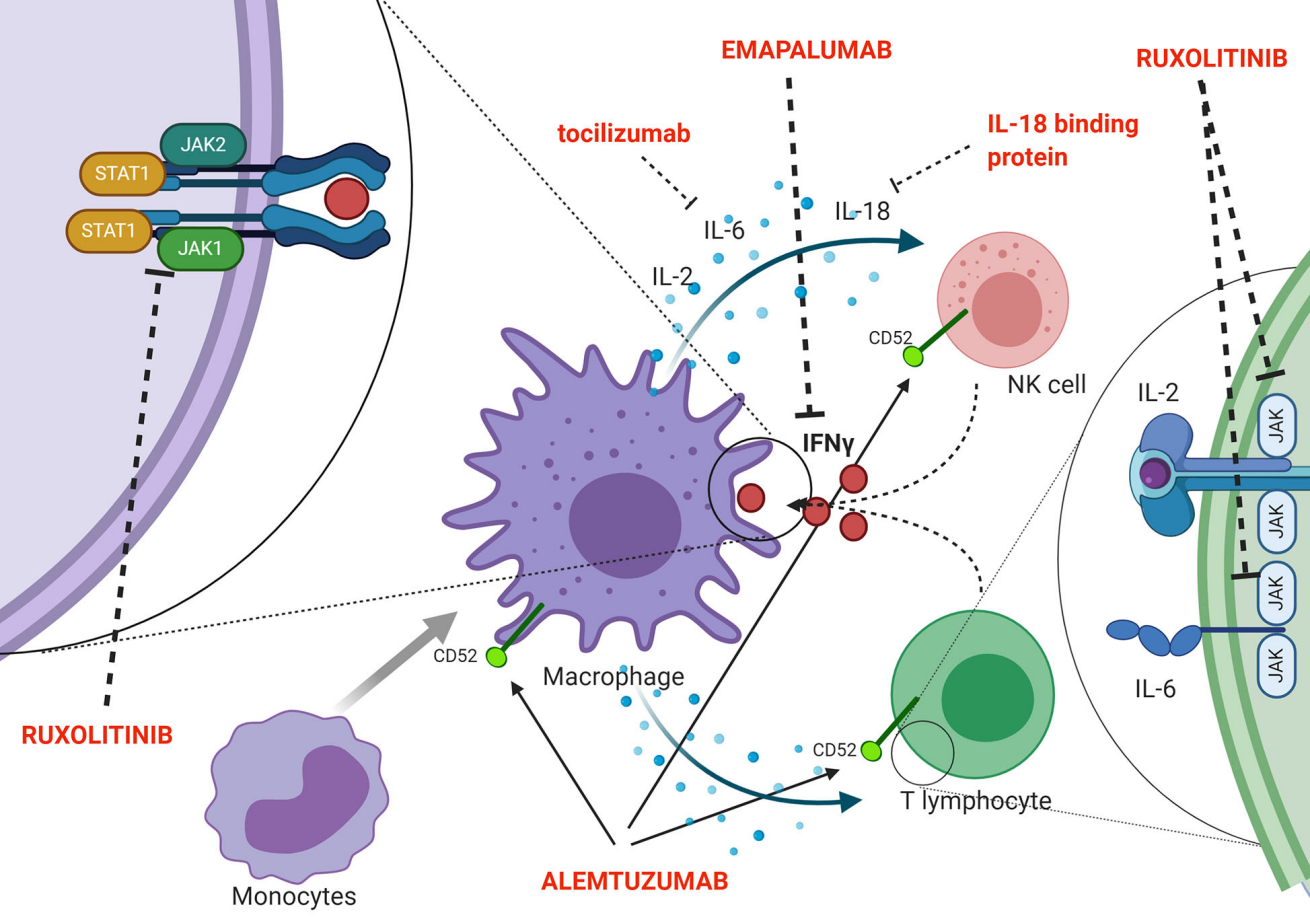
Schematic representation of pathophysiology (including the cytokine storm) and new targeted treatments for HLH.

Indeed, a second effector function of CTLs ensures essential immunoregulation *via* a negative feedback loop, which operates through perforin-dependent killing of antigen-presenting dendritic cells. In pHLH, persistence of activated dendritic cells results in continuous antigen presentation and uncontrolled CTL proliferation (41). Recent evidence suggests that also NK cells exert crucial immunoregulatory functions in the context of pHLH. In particular, models in which cytotoxic defects were restricted to either NK cells or CTLs indicated that functional NK-cell cytotoxicity has a key role in limiting hyperactivation and excessive proliferation of CTLs and it offers protection from severe immunopathology (42). In addition, in murine models, it has been shown that a prolonged synapse time exists between perforin-deficient or granzyme A/B-deficient cytotoxic lymphocytes and target cells. This longer contact results in many successive rounds of Ca2^+^ flux into cytotoxic cells and triggers the overproduction of pro-inflammatory cytokines, further reinforcing the detrimental link between cytotoxicity defects, hypercytokinemia and initiation of systemic inflammation (43). The inability to clear the antigenic stimulus and, thus, to turn off the inflammatory response ultimately leads to a common pathway of uncontrolled cytokine storm, which is responsible for cardinal laboratory and clinical features of HLH. Several potential HLH-promoting cytokines have been identified, including IFNγ, IL-2, TNF-α, IL-6, IL-18, IL-33 (44–48) (**Figure 2** shows a summary of the main cytokines involved in HLH pathogenesis, as well as of new treatment targets). In particular, increasing clinical and experimental evidence suggests that IFNγ plays a crucial role in the pathogenesis of HLH. Serum IFNγ levels and interferon signature are elevated in patients with active disease (44, 48, 49). In a series of 71 patients monitored from HLH diagnosis throughout treatment and follow-up, IFNγ levels above the upper normal limit (17.3 pg/ml) were observed in all patients, with 53.5% having levels above 1,000 pg/ml (48). This can be used, through the use of standardized assays, to facilitate the diagnosis of HLH (50). It was also reported that IFNγ levels rise early and quickly during the course of the disease, and can fall from >5,000 pg/ml to normal in 48 h upon effective treatment of HLH (48). Elevated levels of IFNγ and of the IFNγ-inducible chemokines CXCL9, CXCL10 and CXCL11 were also observed both in patients with HLH secondary to infections and in patients with HLH/MAS occurring in the context of sJIA (49, 51). Noteworthy, levels of IFNγ and IFNγ-inducible chemokines CXCL9 and CXCL10 were significantly higher in patients with MAS as compared with the levels observed in patients with active sJIA without MAS at the time of sampling. In addition, abnormalities in laboratory parameters of MAS, including ferritin, alanine transferase levels, neutrophil and platelet counts, correlated with levels of IFNγ and CXCL9. These findings suggest that IFNγ may also play a pivotal role in the pathophysiology of MAS (49).

IFNγ is a type II interferon secreted by T lymphocytes and NK cells during Th1-mediated immune responses. IFNγ functions as a pro-inflammatory cytokine that mediates antimicrobial, antiviral, and antitumor responses by activating effector immune cells and enhancing antigen presentation (52).

The importance of IFN-γ in the pathogenesis of HLH has been initially strengthened by data obtained in experimental mouse models. It has been demonstrated that, after lymphocytic choriomeningitis virus (LCMV) infection of perforin-deficient mice, hyperactive CTLs and high levels of IFNγ are the driving forces behind the development of fatal HLH (53). In the same model, neutralization of high circulating levels of IFNγ by an anti-mouse IFNγ antibody not only reverted the clinical and laboratory abnormalities, but also dramatically improved animal survival. By contrast, the ablation of other elevated cytokines has no significant impact on mice survival (53).

Similarly, in lymphocytic choriomeningitis virus/Rab27a-deficient mice, neutralization of IFNγ has been shown to revert CNS involvement and to reduce hemophagocytosis (54). Neutralization of IFNγ is also capable to revert hematologic abnormalities in a mouse model of secondary HLH, triggered by repeated stimulation of Toll-like receptor (TLR) 9 on a normal genetic background (55). Finally, in a mouse model of MAS, a significant upregulation of the IFNγ pathway and an increased expression of the IFNγ-inducible chemokines CXCL9 and CXCL10 have been observed in the liver, spleen, and plasma. In the same model, treatment with an anti-IFNγ antibody significantly improved mice survival, reverting clinical and biochemical features of MAS (56).

Hypersecretion of IFNγ may also explain several of the most relevant clinical findings of HLH. In particular, IFNγ was demonstrated to directly stimulate macrophage activation *in vivo*, instigating the onset of hemophagocytosis and possibly causing cytopenias (57). In another experimental model, persistent overexpression of IFNγ was sufficient to induce many of the disease-associated hallmarks associated with autoinflammatory syndromes, including some of those associated with HLH, such as splenomegaly, lymphadenopathy, hyperferritinemia and macrophage activation (58).

Despite this evidence supporting a key role played by IFNγ, the cytokine storm in HLH is quite promiscuous and several other pro-inflammatory mediators might be involved, at different levels, in the immunopathology of the disease.

Recently, IL-33 was demonstrated to be a crucial amplifier of immune dysregulation in murine models of perforin-deficient HLH, being closely linked to IFNγ overproduction. In particular, signalling through the IL-33/ST2 axis promoted CTL activation and production of IFNγ, leading to systemic hypercitokinemia and subsequent lethal inflammation (47).

A discrepancy between the increase in IL-18 and its antagonist IL-18 binding protein (IL-18BP) has been reported in patients with secondary HLH, resulting in aberrantly high levels of free IL-18. High levels of IL-18 induce CTL and macrophage activation, thus contributing to IFNγ production (59, 60). Notably, IL-18 is significantly also elevated in patients with activating lesions of the inflammasome (61). A clinical trial testing the effects of recombinant IL-18 binding protein in patients with NLRC4 Mutation and XIAP Deficiency is currently ongoing (NCT03113760).

Under physiologic conditions, Tregs have a crucial role in peripheral immune tolerance, eliminating activated CTLs in a perforin- and granzyme-dependent way and indirectly suppressing CTL proliferation through preferentially consumption of available IL-2 (62). In perforin-deficient mouse models, limited IL-2 availability was observed, as results of both reduced IL-2 production and elevated levels of antagonistic soluble CD25. The remaining IL-2 was preferably consumed by hyperactivated CTLs, that upregulated their CD25 expression to surpass that of Tregs, inverting the IL2 consumption hierarchy and resulting in a precipitous decrease in Treg cell numbers (63). Recently, the central role of IFNγ in HLH pathogenesis has been challenged by the observation of HLH in IFNγ-deficient mice (64) and in patients with genetic defects in the IFNγ signaling (65). Furthermore, Humblet-Baron and co-workers demonstrated that severe HLH could be induced after LMCV infection in a perforin and IFNγ double knock-out mouse model. In the same model, it was also shown that IFNγ and CD25 pathways act independently of each other in the pathogenesis of HLH, with IFNγ being strictly responsible for hematologic abnormalities, while excessive consumption of IL-2 contributing to immunologic features of HLH (66). These observations reinforce the concept that other mechanisms, beyond IFNγ-driven hyperinflammation, may be involved in HLH pathogenesis and suggest that the therapeutic targeting of these cytokine pathways might provide benefit in the control of the disease.

In this regard, the JAK/STAT pathway is activated by several, pro-inflammatory cytokines that are elevated in HLH and may represent a potential therapeutic target in pHLH. JAK/STAT inhibition with the JAK1/2 inhibitor ruxolitinib has been shown to ameliorate clinical and laboratory manifestations of the disease in Prf1^−/−^ and Rab27α^−/−^ murine models of pHLH, as well as in a mouse model of secondary HLH (67, 68). In particular, Albeituni et al. have demonstrated that in models of both primary and secondary HLH, JAK1/2 inhibition was superior to IFNγ-blocking alone in dampening inflammatory biomarkers, improving clinical parameters and enhancing survival. The beneficial effect of ruxolitinib in these experimental models has been shown to be mediated by both IFNγ-dependent and -independent mechanisms, the latter involving targeting of other cytokines (such as TNF-α and IL-6) and inhibition of T-cell and neutrophil activation and tissue infiltration (69). Preliminary clinical data suggest that ruxolitinib was effective and well tolerated in five patients with secondary HLH (70). Moreover, Wang et al. reported the results obtained with ruxolitinib in 34 patients with refractory/relapsed HLH, the majority (n = 25) being EBV-related. In this cohort, the overall response rate (OR) was 73.5%, with 14.7% (5/34 patients) and 58.8% (20/34 patients) obtaining CR and PR, respectively (71). Recently, Zhang and co-authors treated with ruxolitinib 12 children affected by secondary HLH (mainly Epstein–Barr virus associated HLH). Eight out of 12 patients (66.7%) achieved CR by day 28, while one patient (8.3%) obtained a PR. Notably, no major adverse event was recorded. Considering as event disease progression, relapse or death, with a median follow-up of 8.2 months, the estimated 6-month EFS was 58.3% (72).

## Emapalumab

### Pharmacological Properties

Emapalumab is a fully human monoclonal antibody that works neutralizing both free and receptor-bound IFNγ. Pharmacological properties of the drug are non-linear, being characterized by target-mediated drug disposition (TMDD) phenomenon (https://www.accessdata.fda.gov/drugsatfda_docs/nda/2018/761107Orig1s000MultidisciplineR.pdf). This term refers to a condition in which the pharmacokinetic properties of the drug are influenced by the interactions between the drug itself and its target (e.g., presence of dose-dependent effects on apparent pharmacokinetic parameters, including clearance of the drug, as well as its steady-state volume of distribution). For this reason, pharmacokinetic data differ according to the status of the recipient of the drug. A Phase 1 study performed in the UK in adult healthy subjects (NI-0501-03 study) investigated the safety, tolerability and pharmacokinetic profiles of single i.v. administration of emapalumab: in total, 14 subjects were exposed to increasing doses of emapalumab (ranging from 0.01 to 3 mg/kg). In this healthy population, PK analysis showed a half-life profile expected for an IgG1 (*i.e.*, approximately 22 days).

### Safety and Efficacy—Preclinical

Non-clinical toxicology studies, performed in Cynomolgus monkeys, did not identify developmental or age-specific effects between young and adult animals exposed to emapalumab (https://www.accessdata.fda.gov/drugsatfda_docs/nda/2018/761107Orig1s000MultidisciplineR.pdf).

Since IFNγ is a cytokine with a central role in immune-mediated reaction, its neutralization could be theoretically expected to potentially facilitate the development of infections by specific pathogens including Mycobacteria, Shigella, Campylobacter and Salmonella (73, 74). Accordingly, the use of emapalumab favoured the development of gastrointestinal infections in animals harbouring Shigella, Campylobacter or Salmonella in stools prior the initiation of the drug. Notably, in animals where such pathogens were undetectable before the starting of the treatment, repeated doses of emapalumab were well tolerated without the development of gastrointestinal infections or organ toxicity (https://www.accessdata.fda.gov/drugsatfda_docs/nda/2018/761107Orig1s000MultidisciplineR.pdf).

Patients with pHLH should be evaluated for latent tuberculosis infection with an IFNγ release assay or purified protein derivative (PPD) placement prior to initiation of emapalumab. Patients are also recommended to receive prophylaxis for *Pneumocystis jirovecii*, as well as for varicella-zoster virus (VZV) infection, during the whole duration of therapy with emapalumab.

Preclinical data regarding efficacy have already been discussed above. Noteworthy, emapalumab has been shown to cross react with IFNγ from Rhesus or Cynomolgus monkeys, but not with IFNγ from pig, dog, cat, rat, or mouse. Thus, in preclinical experiments performed in mouse models of HLH, a commercially available anti-mouse IFNγ mAb, XMG1.2, has been extensively used. XMG1.2 is a high-affinity potent inhibitor of IFNγ that has similar functional characteristics to emapalumab, and is deemed a suitable functional surrogate for the conduct of studies that are representative of emapalumab in humans.

### Efficacy—Clinical

Emapalumab efficacy was tested in the NI-0501-04 study (NCT01818492), which enrolled patients aged 0–18 years with a diagnosis of pHLH based on the presence of ≥5 of the eight HLH-2004 diagnostic criteria or genetic confirmation/family history of the disease. The study mainly recruited patients who had failed conventional treatment (e.g., who had received conventional HLH therapy and had a flare of disease after initial response, or who did not achieve a satisfactory response or showing intolerance to the drug) (*i.e.*, second-line patients). Treatment schedule was characterized by an initial dose of emapalumab of 1 mg/kg every 3 days. The dose could be progressively increased up to 10 mg/kg (60). The drug was administered on a backbone of 5–10 mg/m^2^/day of dexamethasone, which could be tapered during the study, with the goal of sparing the steroid-associated toxicity. During emapalumab treatment, intrathecal therapy (glucocorticoids and/or methotrexate) could be administered. Results of NI-0501-04 study were recently published (75). Included in the analysis were 34 patients (out of 53 screened; data cut-off applied was July 2017). Twenty-seven of them were enrolled after failure of first-line treatments, while seven patients were treatment-*naïve*; median age at study entry was 0.85 years (range 0.03–13.0). Twenty-seven out of 34 patients [79%, as compared to approximately 53% of total population of the HLH-2004 protocol (3, 76)] had genetic confirmation of the disease: all forms of FHL were represented, as well as Griscelli syndrome (five patients) and XLP 1 and 2 (one patient each). Notably, 12 patients had signs and/or symptoms of CNS involvement.

The study was completed by 26 patients (76%), while eight (24%) discontinued the study prematurely. A total of 28 patients (82%) entered long-term follow-up (NI-0501-05). The primary endpoint of the study was met, with an overall response rate [ORR (*i.e.*, CR + PR), which was based on pre-defined objective parameters (CR = no fever, a normal spleen size, no cytopenia, no hyperferritinemia, no evidence of coagulopathy, no CNS disease and no sustained increase in the level of sCD25; PR = three or more clinical and laboratory abnormalities that met the criteria for a CR)] at the end of 8-week of treatment of 63% for the previously treated patients (95% CI, 42–81) and 65% for the patients whole population (95% CI, 46 to 80) (p-value 0.02 and 0.005 against pre-specified the null hypothesis of 40%, respectively). Response rate based on investigator’s clinical judgment was 70.6 and 70.4% in the two groups (77). CNS disease was controlled in 6 of the 12 patients with CNS involvement, improved in four, and could not be evaluated in two because of worsening HLH. Six patients received additional treatments (etoposide and/or alemtuzumab) because of an unsatisfactory response based on clinical judgment; all but one were non-responders in the primary efficacy analysis; median exposure to etoposide in patients receiving additional therapy was 450 mg/m^2^, at a median time of 55 days from the start of treatment (76). Among previously treated patients, 26% achieved CR, 30% PR, and 7% had improvement in measures of hemophagocytic lymphohistiocytosis, while 37% had no response. In treatment-naïve patients, 43% achieved PR, 28.5% an improvement and 28.5% no response. Median time to response was 8 days (95% CI 5–10) and it did not differ between first- and second-line patients. Once achieved, the response was maintained for 26 days in 75% of the whole population and for 18 days in 75% of the second-line patients. Notably, low CXCL9 levels were associated with a response in logistic regression analysis, further confirming that neutralization of IFNγ is a valid therapeutic approach. Twenty-four patients were alive at last observation (71%), with an estimated probability of survival of 69.3% (95% CI 50.3–82.2). Twenty-two of them (65%) proceed to HSCT; notably 20 of them are alive after the transplant procedure, with a 1-year estimated probability of survival of 90.2% (95% CI, 66.2 to 97.5). The estimated probability of survival after transplantation at 12 months was 89.5% (95% CI, 64.1 to 97.3) among children who already failed or were intolerant to front-line therapy.

Infusions of emapalumab were well tolerated, with mild to moderate infusion-related reactions reported in 27% of patients. The observed safety events pre-HSCT were represented mostly by HLH manifestations, infections or toxicities due to other administered drugs. Only one patient experienced an infection caused by a pathogen potentially favored by IFNγ neutralization (disseminated histoplasmosis) during emapalumab treatment; however, it resolved without sequelae with appropriate treatment. Notably, no off-target effects were observed (75).

Recently, a case report summarized the successful treatment with emapalumab of a patient with refractory, EBV-associated HLH (78). The patient was a male of Southeast Asian descent who presented at 20 months of age with acute EBV infection and essentially all diagnostic features of HLH as defined by the HLH 2004 criteria. Treatment with emapalumab was initiated after documented failure of HLH conventional therapy (including glucocorticoids and etoposide) along with antiviral (ganciclovir, later switched to brincidofovir for adenoviremia), antifungal (liposomal amphotericin), and antibacterial medications. Treatment of HLH with emapalumab alone resulted in resolution of all clinical symptoms and normalization of clinical laboratory parameters typical of HLH. Treatment was successful despite pre-existing multiple life-threatening infections; in particular, during blockade of IFNγ with emapalumab, all infections resolved with supportive antimicrobial medications and cessation of etoposide and dexamethasone.

Emapalumab was critical to the survival of one patient affected by NOCARH syndrome due to *de novo* CDC42 mutation, who, after stabilization of HLH obtained through the use of the monoclonal antibody, was successfully transplanted from the father (27).

### Use After HSCT

Our group recently reported on three patients affected by pHLH, who, after rejecting a T-cell depleted (TCD) HSCT from an haploidentical donor [both HLH and TCD are well-known risk factors for graft failure (GF) (79)], were treated with emapalumab to successfully control a flare of the disease. Interestingly, patients continued the treatment during and for some weeks after the second HSCT. No significant adverse event was recorded and two out of three patients successfully engrafted. Indeed, in the same manuscript, we showed that GF (especially the primary form) resembles HLH because of clinical findings (*e.g.*, high-grade fever and organomegaly) and cytokine profile (80).

Recently, a child affected by ADA-SCID transplanted from an HLA-haploidentical donor after TCD was successfully treated with emapalumab to control GF-related HLH (81). She continued the treatment during the following transplant in order to prevent GF, with good results. Notably, before starting emapalumab treatment the child had developed several infections (disseminated BCGitis, adenoviral infection, *Stenotrophomonas maltophilia* bacteremia, invasive pulmonary aspergillosis) which did not worsen with IFNγ blockade.

### Other Ongoing Studies on the Use of Emapalumab

Currently, the following studies on the use of emapalumab in different IFNγ-driven conditions are open:

- a Phase 2, single-arm study (a pilot, open-label, single arm, multicenter study to evaluate safety, tolerability, pharmacokinetics and efficacy of intravenous administrations of emapalumab, in patients with systemic Juvenile Idiopathic Arthritis (sJIA) developing Macrophage Activation Syndrome/secondary HLH) in patients with HLH secondary to sJIA, also known as MAS, who have failed to respond to the current standard of care (NI-0501-06 study). The dosing schedule of the drug is quite different from NI-0501-04 study, since emapalumab is administered at the initial dose of 6 and 3 mg/kg twice a week for 4 weeks thereafter. This study will contribute to elucidate the role of IFNγ neutralization in HLH associated with rheumatologic disorders (https://clinicaltrials.gov/ct2/show/NCT03311854). Preliminary, but very promising data on the first six patients have been presented at the 2019 European congress of rheumatology (82). Indeed, emapalumab induced a rapid neutralization of IFNγ and in all six patients control of the disease was achieved by week 8.
- an open-label, single-arm, multicenter interventional study performed in pediatric patients with pHLH (NI-0501-09 study). The study is similar to the NI-0501-04; however, a higher starting dose (i.e., 3 mg/kg) compared to the NI-0501-04 study is being assessed (https://www.clinicaltrialsregister.eu/ctr-search/trial/2017-003114-10/DE);
- an open-label, single-arm, multicenter interventional study conducted in adult patients with secondary HLH (NI-0501-10; ClinicalTrials.gov Identifier: NCT03985423).

## Conclusions

Outcome of pHLH patients remains suboptimal with patients still experiencing high rates of treatment failure and death from different causes. However, advancement in diagnostic algorithm, as well as availability of new drugs (which allows sparing or reduction of cytotoxic drugs), hold the promise of improving patients’ outcome.

Thanks to the phase 2/3 study NI-0501-04 conducted in patients with pHLH (NCT01818492), the neutralization of IFNγ has been validated as a therapeutic target in pHLH. In view of the results of this phase 2/3 study, emapalumab, Gamifant^®^, was approved by the US FDA for adult and pediatric (newborns and older) patients with pHLH with refractory, recurrent or progressive disease or intolerance with conventional HLH therapy. Emapalumab is the first drug approved for the treatment of pHLH with refractory, recurrent or progressive disease, a rare, hyperinflammatory, life-threatening disease. More data are needed to recommend its use in newly diagnosed patients; however, it has to be emphasized that, in light of the failure of HLH-2004 protocol to improve the outcome of pHLH patients in comparison to the HLH-94 study (3), new effective targeted drugs are desirable for the standard treatment of the disease. The need of more effective and safer therapies is further supported by the findings of the recently published study reporting in detail HSCT outcomes of patients enrolled in the HLH-2004 protocol, showing suboptimal results, with a 5-year OS and EFS of 66 and 60%, respectively (83).

The benefit deriving from the addition of drugs conventionally used in the treatment of pHLH (e.g., etoposide ± cyclosporine-A) remains a matter of future investigations through studies designed to address this specific goal.

Given the similarity of pathophysiology of primary and secondary HLH, currently ongoing and future studies will contribute to define the precise role of this IFNγ neutralizing antibody in controlling HLH manifestations occurring in the context of rheumatologic disorders or malignancies.

Finally, based on the effectiveness of emapalumab in inhibiting IFNγ, there is increasing interest in testing the drug in other IFNγ-driven conditions, such as the aforementioned GF occurring after allogeneic HSCT or in patients developing acquired severe aplastic anemia.

As already mentioned, ruxolitinib has shown efficacy in secondary HLH, but its role in pHLH remains to be investigated; several ongoing studies are testing this drug as stand-alone therapy (NCT04120090) or in combination [(NCT04551131) and (NCT03533790)] for newly diagnosed and/or relapsed/refractory pHLH. A comparison of the benefits and risks associated with either emapalumab or ruxolitinib and their respective role in pHLH management will be defined in view of the data that will become available in the next years. For the time being, we can just valorize that ruxolitinib has the potential of pleiotropically block the transduction pathway of several inflammatory cytokines, but its use has been reported to be associated with hematological cytopenia and, in patients with acute GVHD, with an increased risk of CMV infections (84).

## Author Contributions

All authors contributed to the article and approved the submitted version.

## Conflict of Interest

PM and FL received consulting fees from Sobi.

The remaining authors declare that the research was conducted in the absence of any commercial or financial relationships that could be construed as a potential conflict of interest.

## References

[1] Janka GE Familial hemophagocytic lymphohistiocytosis. Eur J Pediatr (1983) 140:221–30. 10.1007/bf00443367 6354720

[2] Trottestam H Horne A Arico M Egeler RM Filipovich AH Gadner H Chemoimmunotherapy for hemophagocytic lymphohistiocytosis: long-term results of the HLH-94 treatment protocol. Blood (2011) 118:4577–84. 10.1182/blood-2011-06-356261 PMC3208276 21900192

[3] Bergsten E Horne A Arico M Astigarraga I Egeler RM Filipovich AH Confirmed efficacy of etoposide and dexamethasone in HLH treatment: long-term results of the cooperative HLH-2004 study. Blood (2017) 130:2728–38. 10.1182/blood-2017-06-788349 PMC5785801 28935695

[4] Marsh RA Allen CE McClain KL Weinstein JL Kanter J Skiles J Salvage therapy of refractory hemophagocytic lymphohistiocytosis with alemtuzumab. Pediatr Blood Cancer (2013) 60:101–9. 10.1002/pbc.24188 PMC3410971 22522603

[5] Mahlaoui N Ouachee-Chardin M de Saint Basile G Neven B Picard C Blanche S Immunotherapy of familial hemophagocytic lymphohistiocytosis with antithymocyte globulins: a single-center retrospective report of 38 patients. Pediatrics (2007) 120:e622–8. 10.1542/peds.2006-3164 17698967

[6] Marsh RA Jordan MB Talano JA Nichols KE Kumar A Naqvi A Salvage therapy for refractory hemophagocytic lymphohistiocytosis: A review of the published experience. Pediatr Blood Cancer (2017) 64(4):e26308. 10.1002/pbc.26308 27786410

[7] Broglie L Pommert L Rao S Thakar M Phelan R Margolis D Ruxolitinib for treatment of refractory hemophagocytic lymphohistiocytosis. Blood Adv (2017) 1:1533–6. 10.1182/bloodadvances.2017007526 PMC5728466 29296794

[8] Stepp SE Dufourcq-Lagelouse R Le Deist F Bhawan S Certain S Mathew PA Perforin gene defects in familial hemophagocytic lymphohistiocytosis. Science (1999) 286:1957–9. 10.1126/science.286.5446.1957 10583959

[9] Janka GE Lehmberg K Hemophagocytic lymphohistiocytosis: pathogenesis and treatment. Hematol Am Soc Hematol Educ Program (2013) 2013:605–11. 10.1182/asheducation-2013.1.605 24319239

[10] Sepulveda FE de Saint Basile G Hemophagocytic syndrome: primary forms and predisposing conditions. Curr Opin Immunol (2017) 49:20–6. 10.1016/j.coi.2017.08.004 28866302

[11] de Saint Basile G Menasche G Fischer A Molecular mechanisms of biogenesis and exocytosis of cytotoxic granules. Nat Rev Immunol (2010) 10:568–79. 10.1038/nri2803 20634814

[12] Lopez JA Susanto O Jenkins MR Lukoyanova N Sutton VR Law RH Perforin forms transient pores on the target cell plasma membrane to facilitate rapid access of granzymes during killer cell attack. Blood (2013) 121:2659–68. 10.1182/blood-2012-07-446146 23377437

[13] Cote M Menager MM Burgess A Mahlaoui N Picard C Schaffner C Munc18-2 deficiency causes familial hemophagocytic lymphohistiocytosis type 5 and impairs cytotoxic granule exocytosis in patient NK cells. J Clin Invest (2009) 119:3765–73. 10.1172/JCI40732 PMC2786810 19884660

[14] Feldmann J Callebaut I Raposo G Certain S Bacq D Dumont C Munc13-4 is essential for cytolytic granules fusion and is mutated in a form of familial hemophagocytic lymphohistiocytosis (FHL3). Cell (2003) 115:461–73. 10.1016/S0092-8674(03)00855-9 14622600

[15] zur Stadt U Rohr J Seifert W Koch F Grieve S Pagel J Familial hemophagocytic lymphohistiocytosis type 5 (FHL-5) is caused by mutations in Munc18-2 and impaired binding to syntaxin 11. Am J Hum Genet (2009) 85:482–92. 10.1016/j.ajhg.2009.09.005 PMC2756548 19804848

[16] zur Stadt U Schmidt S Kasper B Beutel K Diler AS Henter JI Linkage of familial hemophagocytic lymphohistiocytosis (FHL) type-4 to chromosome 6q24 and identification of mutations in syntaxin 11. Hum Mol Genet (2005) 14:827–34. 10.1093/hmg/ddi076 15703195

[17] Pagel J Beutel K Lehmberg K Koch F Maul-Pavicic A Rohlfs AK Distinct mutations in STXBP2 are associated with variable clinical presentations in patients with familial hemophagocytic lymphohistiocytosis type 5 (FHL5). Blood (2012) 119:6016–24. 10.1182/blood-2011-12-398958 22451424

[18] Enders A Zieger B Schwarz K Yoshimi A Speckmann C Knoepfle EM Lethal hemophagocytic lymphohistiocytosis in Hermansky-Pudlak syndrome type II. Blood (2006) 108:81–7. 10.1182/blood-2005-11-4413 16551969

[19] Menasche G Pastural E Feldmann J Certain S Ersoy F Dupuis S Mutations in RAB27A cause Griscelli syndrome associated with haemophagocytic syndrome. Nat Genet (2000) 25:173–6. 10.1038/76024 10835631

[20] Nagle DL Karim MA Woolf EA Holmgren L Bork P Misumi DJ Identification and mutation analysis of the complete gene for Chediak-Higashi syndrome. Nat Genet (1996) 14:307–11. 10.1038/ng1196-307 8896560

[21] Pachlopnik Schmid J Canioni D Moshous D Touzot F Mahlaoui N Hauck F Clinical similarities and differences of patients with X-linked lymphoproliferative syndrome type 1 (XLP-1/SAP deficiency) versus type 2 (XLP-2/XIAP deficiency). Blood (2011) 117:1522–9. 10.1182/blood-2010-07-298372 21119115

[22] Sayos J Wu C Morra M Wang N Zhang X Allen D The X-linked lymphoproliferative-disease gene product SAP regulates signals induced through the co-receptor SLAM. Nature (1998) 395:462–9. 10.1038/26683 9774102

[23] Canna SW de Jesus AA Gouni S Brooks SR Marrero B Liu Y An activating NLRC4 inflammasome mutation causes autoinflammation with recurrent macrophage activation syndrome. Nat Genet (2014) 46:1140–6. 10.1038/ng.3089 PMC4177369 25217959

[24] Romberg N Al Moussawi K Nelson-Williams C Stiegler AL Loring E Choi M Mutation of NLRC4 causes a syndrome of enterocolitis and autoinflammation. Nat Genet (2014) 46:1135–9. 10.1038/ng.3066 PMC4177367 25217960

[25] Marsh RA Madden L Kitchen BJ Mody R McClimon B Jordan MB XIAP deficiency: a unique primary immunodeficiency best classified as X-linked familial hemophagocytic lymphohistiocytosis and not as X-linked lymphoproliferative disease. Blood (2010) 116:1079–82. 10.1182/blood-2010-01-256099 PMC2938130 20489057

[26] Bode SF Ammann S Al-Herz W Bataneant M Dvorak CC Gehring S The syndrome of hemophagocytic lymphohistiocytosis in primary immunodeficiencies: implications for differential diagnosis and pathogenesis. Haematologica (2015) 100:978–88. 10.3324/haematol.2014.121608 PMC4486233 26022711

[27] Lam MT Coppola S Krumbach OHF Prencipe G Insalaco A Cifaldi C A novel disorder involving dyshematopoiesis, inflammation, and HLH due to aberrant CDC42 function. J Exp Med (2019) 216:2778–99. 10.1084/jem.20190147 PMC6888978 31601675

[28] Zhang K Jordan MB Marsh RA Johnson JA Kissell D Meller J Hypomorphic mutations in PRF1, MUNC13-4, and STXBP2 are associated with adult-onset familial HLH. Blood (2011) 118:5794–8. 10.1182/blood-2011-07-370148 PMC3228496 21881043

[29] Brisse E Wouters CH Matthys P Advances in the pathogenesis of primary and secondary haemophagocytic lymphohistiocytosis: differences and similarities. Br J Haematol (2016) 174:203–17. 10.1111/bjh.14147 27264204

[30] Spinner MA Ker JP Stoudenmire CJ Fadare O Mace EM Orange JS GATA2 deficiency underlying severe blastomycosis and fatal herpes simplex virus-associated hemophagocytic lymphohistiocytosis. J Allergy Clin Immunol (2016) 137:638–40. 10.1016/j.jaci.2015.07.043 PMC4747814 26395816

[31] Singh G Shabani-Rad MT Vanderkooi OG Vayalumkal JV Kuhn SM Guilcher GM Leishmania in HLH: a rare finding with significant treatment implications. J Pediatr Hematol Oncol (2013) 35:e127–9. 10.1097/MPH.0b013e318286d619 23511497

[32] Jordan MB Allen CE Weitzman S Filipovich AH McClain KL How I treat hemophagocytic lymphohistiocytosis. Blood (2011) 118:4041–52. 10.1182/blood-2011-03-278127 PMC3204727 21828139

[33] Atteritano M David A Bagnato G Beninati C Frisina A Iaria C Haemophagocytic syndrome in rheumatic patients. A systematic review. Eur Rev Med Pharmacol Sci (2012) 16:1414–24. 23104659

[34] Zhang K Biroschak J Glass DN Thompson SD Finkel T Passo MH Macrophage activation syndrome in patients with systemic juvenile idiopathic arthritis is associated with MUNC13-4 polymorphisms. Arthritis Rheum (2008) 58:2892–6. 10.1002/art.23734 PMC2779064 18759271

[35] Lehmberg K Sprekels B Nichols KE Woessmann W Muller I Suttorp M Malignancy-associated haemophagocytic lymphohistiocytosis in children and adolescents. Br J Haematol (2015) 170:539–49. 10.1111/bjh.13462 25940575

[36] Booth C Gilmour KC Veys P Gennery AR Slatter MA Chapel H X-linked lymphoproliferative disease due to SAP/SH2D1A deficiency: a multicenter study on the manifestations, management and outcome of the disease. Blood (2011) 117:53–62. 10.1182/blood-2010-06-284935 20926771 PMC3374620

[37] Clementi R Locatelli F Dupre L Garaventa A Emmi L Bregni M A proportion of patients with lymphoma may harbor mutations of the perforin gene. Blood (2005) 105:4424–8. 10.1182/blood-2004-04-1477 15728124

[38] Janka GE Lehmberg K Hemophagocytic syndromes–an update. Blood Rev (2014) 28:135–42. 10.1016/j.blre.2014.03.002 24792320

[39] Chen J Feng X Desierto MJ Keyvanfar K Young NS IFN-gamma-mediated hematopoietic cell destruction in murine models of immune-mediated bone marrow failure. Blood (2015) 126:2621–31. 10.1182/blood-2015-06-652453 PMC4671109 26491068

[40] Heeg M Ammann S Klemann C Panning M Falcone V Hengel H Is an infectious trigger always required for primary hemophagocytic lymphohistiocytosis? Lessons from in utero and neonatal disease. Pediatr Blood Cancer (2018) 65:e27344. 10.1002/pbc.27344 30070073 PMC7168068

[41] Terrell CE Jordan MB Perforin deficiency impairs a critical immunoregulatory loop involving murine CD8(+) T cells and dendritic cells. Blood (2013) 121:5184–91. 10.1182/blood-2013-04-495309 PMC3695362 23660960

[42] Sepulveda FE Maschalidi S Vosshenrich CA Garrigue A Kurowska M Menasche G A novel immunoregulatory role for NK-cell cytotoxicity in protection from HLH-like immunopathology in mice. Blood (2015) 125:1427–34. 10.1182/blood-2014-09-602946 25525117

[43] Jenkins MR Rudd-Schmidt JA Lopez JA Ramsbottom KM Mannering SI Andrews DM Failed CTL/NK cell killing and cytokine hypersecretion are directly linked through prolonged synapse time. J Exp Med (2015) 212:307–17. 10.1084/jem.20140964 PMC4354371 25732304

[44] Henter JI Elinder G Soder O Hansson M Andersson B Andersson U Hypercytokinemia in familial hemophagocytic lymphohistiocytosis. Blood (1991) 78:2918–22. 10.1182/blood.V78.11.2918.bloodjournal78112918 1954380

[45] Osugi Y Hara J Tagawa S Takai K Hosoi G Matsuda Y Cytokine production regulating Th1 and Th2 cytokines in hemophagocytic lymphohistiocytosis. Blood (1997) 89:4100–3. 10.1182/blood.V89.11.4100 9166851

[46] Put K Avau A Brisse E Mitera T Put S Proost P Cytokines in systemic juvenile idiopathic arthritis and haemophagocytic lymphohistiocytosis: tipping the balance between interleukin-18 and interferon-gamma. Rheumatol (Oxford) (2015) 54:1507–17. 10.1093/rheumatology/keu524 25767156

[47] Rood JE Rao S Paessler M Kreiger PA Chu N Stelekati E ST2 contributes to T-cell hyperactivation and fatal hemophagocytic lymphohistiocytosis in mice. Blood (2016) 127:426–35. 10.1182/blood-2015-07-659813 PMC4731846 26518437

[48] Xu XJ Tang YM Song H Yang SL Xu WQ Zhao N Diagnostic accuracy of a specific cytokine pattern in hemophagocytic lymphohistiocytosis in children. J Pediatr (2012) 160:984–90 e1. 10.1016/j.jpeds.2011.11.046 22226576

[49] Bracaglia C de Graaf K Pires Marafon D Guilhot F Ferlin W Prencipe G Elevated circulating levels of interferon-gamma and interferon-gamma-induced chemokines characterise patients with macrophage activation syndrome complicating systemic juvenile idiopathic arthritis. Ann Rheum Dis (2017) 76:166–72. 10.1136/annrheumdis-2015-209020 27296321

[50] Merli P Gentile L Quagliarella F Cefalo MG Strocchio L Locatelli F QuantiFERON-TB Gold can help clinicians in the diagnosis of haemophagocytic lymphohistiocytosis. Br J Haematol (2020) 191:e41–69. 10.1111/bjh.17001 32712963

[51] Buatois V Chatel L Cons L Lory S Richard F Guilhot F Use of a mouse model to identify a blood biomarker for IFNgamma activity in pediatric secondary hemophagocytic lymphohistiocytosis. Transl Res (2017) 180:37–52 e2. 10.1016/j.trsl.2016.07.023 27559680 PMC7185816

[52] Schroder K Hertzog PJ Ravasi T Hume DA Interferon-gamma: an overview of signals, mechanisms and functions. J Leukoc Biol (2004) 75:163–89. 10.1189/jlb.0603252 14525967

[53] Jordan MB Hildeman D Kappler J Marrack P An animal model of hemophagocytic lymphohistiocytosis (HLH): CD8+ T cells and interferon gamma are essential for the disorder. Blood (2004) 104:735–43. 10.1182/blood-2003-10-3413 15069016

[54] Pachlopnik Schmid J Ho CH Chretien F Lefebvre JM Pivert G Kosco-Vilbois M Neutralization of IFNgamma defeats haemophagocytosis in LCMV-infected perforin- and Rab27a-deficient mice. EMBO Mol Med (2009) 1:112–24. 10.1002/emmm.200900009 PMC3378118 20049711

[55] Behrens EM Canna SW Slade K Rao S Kreiger PA Paessler M Repeated TLR9 stimulation results in macrophage activation syndrome-like disease in mice. J Clin Invest (2011) 121:2264–77. 10.1172/JCI43157 PMC3104738 21576823

[56] Prencipe G Caiello I Pascarella A Grom AA Bracaglia C Chatel L Neutralization of IFN-gamma reverts clinical and laboratory features in a mouse model of macrophage activation syndrome. J Allergy Clin Immunol (2018) 141:1439–49. 10.1016/j.jaci.2017.07.021 28807602

[57] Zoller EE Lykens JE Terrell CE Aliberti J Filipovich AH Henson PM Hemophagocytosis causes a consumptive anemia of inflammation. J Exp Med (2011) 208:1203–14. 10.1084/jem.20102538 PMC3173248 21624938

[58] Lee Reinhardt R Liang HE Bao K Price AE Mohrs M Kelly BL A novel model for IFN-gamma-mediated autoinflammatory syndromes. J Immunol (2015) 194:2358–68. 10.4049/jimmunol.1401992 PMC4337485 25637019

[59] Mazodier K Marin V Novick D Farnarier C Robitail S Schleinitz N Severe imbalance of IL-18/IL-18BP in patients with secondary hemophagocytic syndrome. Blood (2005) 106:3483–9. 10.1182/blood-2005-05-1980 PMC1895045 16020503

[60] Girard-Guyonvarc’h C Palomo J Martin P Rodriguez E Troccaz S Palmer G Unopposed IL-18 signaling leads to severe TLR9-induced macrophage activation syndrome in mice. Blood (2018) 131:1430–41. 10.1182/blood-2017-06-789552 29295842

[61] Weiss ES Girard-Guyonvarc’h C Holzinger D de Jesus AA Tariq Z Picarsic J Interleukin-18 diagnostically distinguishes and pathogenically promotes human and murine macrophage activation syndrome. Blood (2018) 131:1442–55. 10.1182/blood-2017-12-820852 PMC5877443 29326099

[62] Kastenmuller W Gasteiger G Subramanian N Sparwasser T Busch DH Belkaid Y Regulatory T cells selectively control CD8+ T cell effector pool size via IL-2 restriction. J Immunol (2011) 187:3186–97. 10.4049/jimmunol.1101649 PMC3169715 21849683

[63] Humblet-Baron S Franckaert D Dooley J Bornschein S Cauwe B Schonefeldt S IL-2 consumption by highly activated CD8 T cells induces regulatory T-cell dysfunction in patients with hemophagocytic lymphohistiocytosis. J Allergy Clin Immunol (2016) 138:200–9 e8. 10.1016/j.jaci.2015.12.1314 26947179

[64] Brisse E Imbrechts M Put K Avau A Mitera T Berghmans N Mouse Cytomegalovirus Infection in BALB/c Mice Resembles Virus-Associated Secondary Hemophagocytic Lymphohistiocytosis and Shows a Pathogenesis Distinct from Primary Hemophagocytic Lymphohistiocytosis. J Immunol (2016) 196:3124–34. 10.4049/jimmunol.1501035 26903481

[65] Tesi B Sieni E Neves C Romano F Cetica V Cordeiro AI Hemophagocytic lymphohistiocytosis in 2 patients with underlying IFN-gamma receptor deficiency. J Allergy Clin Immunol (2015) 135:1638–41. 10.1016/j.jaci.2014.11.030 25592983

[66] Humblet-Baron S Franckaert D Dooley J Ailal F Bousfiha A Deswarte C IFN-gamma and CD25 drive distinct pathologic features during hemophagocytic lymphohistiocytosis. J Allergy Clin Immunol (2018) 143(6):2215–26.e7. 10.1016/j.jaci.2018.10.068 PMC7117880 30578871

[67] Das R Guan P Sprague L Verbist K Tedrick P An QA Janus kinase inhibition lessens inflammation and ameliorates disease in murine models of hemophagocytic lymphohistiocytosis. Blood (2016) 127:1666–75. 10.1182/blood-2015-12-684399 PMC4817310 26825707

[68] Maschalidi S Sepulveda FE Garrigue A Fischer A de Saint Basile G Therapeutic effect of JAK1/2 blockade on the manifestations of hemophagocytic lymphohistiocytosis in mice. Blood (2016) 128:60–71. 10.1182/blood-2016-02-700013 27222478

[69] Albeituni S Verbist KC Tedrick PE Tillman H Picarsic J Bassett R Mechanisms of action of ruxolitinib in murine models of hemophagocytic lymphohistiocytosis. Blood (2019) 134:147–59. 10.1182/blood.2019000761 PMC6624972 31015190

[70] Ahmed A Merrill SA Alsawah F Bockenstedt P Campagnaro E Devata S Ruxolitinib in adult patients with secondary haemophagocytic lymphohistiocytosis: an open-label, single-centre, pilot trial. Lancet Haematol (2019) 6:e630–e7. 10.1016/S2352-3026(19)30156-5 PMC8054981 31537486

[71] Wang J Wang Y Wu L Wang X Jin Z Gao Z Ruxolitinib for refractory/relapsed hemophagocytic lymphohistiocytosis. Haematologica (2020) 105:e210–e2. 10.3324/haematol.2019.222471 PMC7193462 31515353

[72] Zhang Q Wei A Ma HH Zhang L Lian HY Wang D A pilot study of ruxolitinib as a front-line therapy for 12 children with secondary hemophagocytic lymphohistiocytosis. Haematologica (2020). 10.3324/haematol.2020.253781 PMC8252948 32732367

[73] Edwards LA Nistala K Mills DC Stephenson HN Zilbauer M Wren BW Delineation of the innate and adaptive T-cell immune outcome in the human host in response to Campylobacter jejuni infection. PloS One (2010) 5:e15398. 10.1371/journal.pone.0015398 21085698 PMC2976761

[74] Le-Barillec K Magalhaes JG Corcuff E Thuizat A Sansonetti PJ Phalipon A and NK cells in the innate immune response to Shigella flexneri. J Immunol (2005) 175:1735–40. 10.4049/jimmunol.175.3.1735 16034114

[75] Locatelli F Jordan MB Allen C Cesaro S Rizzari C Rao A Emapalumab in Children with Primary Hemophagocytic Lymphohistiocytosis. N Engl J Med (2020) 382:1811–22. 10.1056/NEJMoa1911326 32374962

[76] Jordan MB Locatelli F Emapalumab in Primary Hemophagocytic Lymphohistiocytosis. Reply. N Engl J Med (2020) 383:598–9. 10.1056/NEJMc2020754 32757535

[77] Locatelli F Jordan MB Allen CE Cesaro S Rizzari C Rao A Safety and Efficacy of Emapalumab in Pediatric Patients with Primary Hemophagocytic Lymphohistiocytosis. Blood (2018) 132:3. 10.1182/blood-2018-120810 29976777

[78] Lounder DT Bin Q de Min C Jordan MB Treatment of refractory hemophagocytic lymphohistiocytosis with emapalumab despite severe concurrent infections. Blood Adv (2019) 3:47–50. 10.1182/bloodadvances.2018025858 30617216 PMC6325304

[79] Locatelli F Lucarelli B Merli P Current and future approaches to treat graft failure after allogeneic hematopoietic stem cell transplantation. Expert Opin Pharmacother (2014) 15:23–36. 10.1517/14656566.2014.852537 24156789

[80] Merli P Caruana I De Vito R Strocchio L Weber G Del Bufalo F Role of IFNgamma in immune-mediated graft failure occurring after allogeneic hematopoietic stem cell transplantation. Haematologica (2019) 104(11)2314-23. 10.3324/haematol.2019.216101 PMC6821635 30792213

[81] Tucci F Gallo V Barzaghi F Ferrua F Migliavacca M Calbi V Treatment with emapalumab in an ADA-SCID patient with refractory hemophagocytic lymphohistiocytosis-related graft failure and disseminated BCGitis. Haematologica (2020). 10.3324/haematol.2020.255620 PMC7849754 32817285

[82] De Benedetti F Brogan P Grom A Quartier P Schneider R De Graaf K EMAPALUMAB, AN INTERFERON GAMMA (IFN-Y)-BLOCKING MONOCLONAL ANTIBODY, IN PATIENTS WITH MACROPHAGE ACTIVATION SYNDROME (MAS) COMPLICATING SYSTEMIC JUVENILE IDIOPATHIC ARTHRITIS (SJIA). Ann Rheum Dis (2019) 78:178–. 10.1136/annrheumdis-2019-eular.3341

[83] Bergsten E Horne A Hed Myrberg I Arico M Astigarraga I Ishii E Stem cell transplantation for children with hemophagocytic lymphohistiocytosis: results from the HLH-2004 study. Blood Adv (2020) 4:3754–66. 10.1182/bloodadvances.2020002101 PMC7422132 32780845

[84] Zeiser R von Bubnoff N Butler J Mohty M Niederwieser D Or R Ruxolitinib for Glucocorticoid-Refractory Acute Graft-versus-Host Disease. N Engl J Med (2020) 382:1800–10. 10.1056/NEJMoa1917635 32320566

